# Label-free quantitative proteomic analysis of serum exosomes in mice with thoracic aortic aneurysm

**DOI:** 10.1186/s12953-023-00220-x

**Published:** 2023-10-24

**Authors:** Jia Xu, Jiacheng Liu, Yibai Qu, Linhui Jiang, Rongxin Liang, Bohai Li, Lei Li, Yong Jiang

**Affiliations:** 1 Department of Anesthesiology, The Third Affiliated Hospital of Southern Medical University, Guangzhou, 510630 Guangdong China; 2 Department of Cardiovascular Surgery, Affiliated Guangdong Second Provincial General Hospital, Jinan University, Guangzhou, 510000 Guangdong China; 3 Guangdong Provincial Key Laboratory of Proteomics, State Key Laboratory of Organ Failure Research, Department of Pathophysiology, School of Basic Medical Sciences, Southern Medical University, Guangzhou, 510000 Guangdong China; 4 Guangdong Provincial People’s Hospital (Guangdong Academy of Medical Sciences), Southern Medical University, Guangzhou, 510000 Guangdong China; 5 Department of Neurology, Shenzhen Hospital of Southern Medical University, Shenzhen, 518000 Guangdong China

**Keywords:** Thoracic aortic aneurysm, β-aminopropionitrile, Angiotensin II, Proteomics

## Abstract

**Objective:**

Thoracic aortic aneurysm (TAA) is a cardiovascular disease with high morbidity and mortality. However, the causes and mechanisms of TAA are not fully understood. Serum exosomes from mice with TAA were used to explore the markers associated with this disease.

**Methods:**

C57BL/6 mice were divided into three groups and given ordinary drinking water, ordinary drinking water plus a saline osmotic pump, or drinking water containing β-aminopropionitrile (BAPN) (1 g/kg/d) plus an angiotensin II (Ang II) (1 μg/kg/min) osmotic pump. Haematoxylin and eosin staining of thoracic aortic tissues was performed. The basic characteristics of exosomes were analysed. Differentially expressed proteins (DEPs) were identified by LC‒MS/MS. Protein‒protein networks and enrichment analysis were used to explore possible molecular mechanisms.

**Results:**

The present study elucidated the protein expression profile of serum exosomes in mice with TAA induced by BAPN combined with Ang II. In this work, the expression of a total of 196 proteins was significantly dysregulated in serum exosomes of mice with TAA, with 122 proteins significantly upregulated and 74 proteins markedly downregulated. Notably, Haptoglobin (Hp) and Serum amyloid p-component (Sap) identified based on the PPI network were significantly upregulated and have been strongly linked to cardiovascular disease. Interestingly, Kyoto Encyclopedia of Genes and Genomes (KEGG) pathway analysis showed that the upregulated and downregulated proteins were involved in the complement and coagulation cascade pathways.

**Conclusions:**

This study showed that the identified DEPs have potential as biomarkers for the diagnosis of TAA and provided a more comprehensive understanding of the pathophysiological mechanisms of TAA.

**Supplementary Information:**

The online version contains supplementary material available at 10.1186/s12953-023-00220-x.

## Introduction

Thoracic aortic aneurysm (TAA) is an acute cardiovascular disease with high mortality, with an incidence of 7.6 per 100,000 in Western countries [1]. TAA is considered a common cause of death in elderly individuals [2]. During TAA progression, the switch from a contractile to a synthetic vSMC phenotype is induced by inflammatory cell infiltration, oxidative stress, and mechanical wall stress, contributing to increased proteolytic enzyme production. These proteolytic enzymes promote extracellular matrix (Ecm) degradation, facilitating vSMC apoptosis and dilating the aortic wall [3].

The diagnosis of TAA mainly depends on computed tomography angiography (CTA), magnetic resonance imaging (MRI), and echocardiography [4]. However, TAA is not fully dilated in the early phase of the disease, so the diagnostic efficacy of those techniques at that stage is not well. Balmforth et al. reported increased levels of D-dimer, susceptible C-reactive protein, and homocysteine in the serum of aortic aneurysm patients [5]. However, these indicators have increased levels in various diseases and cannot be effectively used as biomarkers for diagnosing TAA due to their low specificity. Therefore, it is necessary to predict aneurysm progression by exploring specific serum biomarkers [6].

Exosomes are vesicles with a bilayer membranous structure that are secreted by various cells and are approximately 30–150 nm in diameter [7]. They have been found in various body fluids, including blood, saliva, urine, cerebrospinal fluid, and breast milk. Although exosomes used to be considered cellular waste, recent research shows that lncRNAs can regulate important cellular physiological activities by carrying related proteins, mRNAs, long-chain noncoding RNAs (lncRNAs), and miRNAs [8]. Therefore, exosomes are biomarkers that can be used for disease diagnosis and have great potential for clinical applications.

In recent years, interest in exosome research has increased due to their potential for clinical application in diagnosing and treating disease. Su et al. have shown that exosomes are secreted by many cells, such as endothelial cells, vascular smooth muscle cells, and mesenchymal stem cells [9]. Likewise, research has revealed that the level of miRNA in the circulation is directly connected to the shear force along the vessel walls among patients with bicuspid aortic valve ascending aortic aneurysm (BAV AsAA) [10]. It has been reported that the progression of aortic aneurysm dilation induced by calcium phosphate (CaPO_4_) is associated with exosome release in the plasma. The incidence of aortic aneurysms in mice was significantly reduced following intraperitoneal injection of the exosome biogenesis inhibitor GW4869 (a neutral sphingomyelinase inhibitor) [11]. These studies showed that exosomes are closely associated with the progression of aortic aneurysms.

In this study, a murine model of TAA was induced via the administration of β-aminopropionitrile (BAPN), a lysine oxidase inhibitor, combined with a continuous subcutaneous infusion of Ang II [12]. To analyse the changes in the protein profiles of serum exosomes in a murine model of TAA, we used liquid chromatography‒mass spectrometry (LC‒MS/MS). Our findings provide a theoretical basis for understanding the pathogenesis of TAA and improving strategies for the diagnosis and treatment of the disease.

## Materials and methods

### Animals

Wild-type C57BL/6 male mice (3 weeks old, *n* = 108) with similar body weights were purchased from the Laboratory Animal Center of Southern Medical University. All mice were maintained under standard environmental conditions at room temperature. The Animal Ethical and Welfare Committee of Southern Medical University reviewed and approved all animal procedures and protocols.

### Construction of animal models of TAA

Wild-type male C57BL/6 mice with similar body weights at 3 weeks of age were divided into three groups: the control group (*n* = 36, ordinary drinking water), sham group (*n* = 36, ordinary drinking water plus a saline osmotic pump), and model group (*n* = 36, drinking water containing BAPN plus an Ang II osmotic pump) [12]. Before the experiment, the mice were fed an unrestricted diet for 2 days, and water intake and weight were recorded. For each group of mice, a high-frequency ultrasonic imaging system was used to measure the diameter of the thoracic aorta (at the midpoint of the ascending aorta) and abdominal aorta (at the midpoint between the coeliac trunk artery and renal artery). For each mouse, these parameters were measured 3 times, and the average value was used. After two days, we determined the dose of BAPN (Sigma‒Aldrich, USA) according to diet and water intake per cage. The mice were administered BAPN in the drinking water at 1 g/kg/d for 28 days. After the mice were given drinking water containing BAPN for 28 days, a micro-osmotic pump (Alzet, USA) that released Ang II (Sigma‒Aldrich, USA) at a rate of 1 μg/kg/min was implanted into model group mice on a sterile operating table under intraperitoneal anaesthesia with 1% sodium pentobarbital. The same method was used to implant a micro-osmotic pump containing physiological saline into the mice in the sham group. One day before surgery, we placed the micro-osmotic pumps in a sterile petri dish with physiological saline and incubated them in a cell culture incubator at 37 °C for 24 h. After the operation, the survival of the mice was monitored every 2 h until the end of the experiment.

### Baseline characterization of the mice and aortic morphology

The hair colour and mental status of the mice were observed daily. Weight and water intake were monitored during the establishment of the murine model of TAA. The cause of death was defined by dissection for mice that died before the expected end point of the experiment. After forty-eight hours of implanting the micro-osmotic pump, the diameter of the thoracic and abdominal aorta of surviving mice was measured with a high-frequency ultrasonic imaging system under intraperitoneal anaesthesia with 1% sodium pentobarbital, and each mouse was measured three times. The average value was taken. Blood was collected from the inferior vena cava, the aorta was separated from other tissues under a stereo microscope, and the aorta tissues were fixed with 4% paraformaldehyde.

### Haematoxylin–eosin (HE) staining

The aortic tissues were fixed with 4% paraformaldehyde and incubated at room temperature with shaking for 48 h. Afterwards, the aortic tissues were submerged in ethanol at different concentrations for 2 h for gradient dehydration and embedded in paraffin. The specimens were sectioned into 5 μm sections and baked at 70 °C for 30 min. The sections were dewaxed in xylene and rehydrated. The sections were stained with haematoxylin for 6 min, stained with eosin for 2 min and reimmersed in ethanol and xylene for dehydration and clearing. The slides were mounted with glycerin resin and observed under a microscope.

### Exosome extraction and isolation

Venous blood was allowed to stand for 30 min at room temperature and then centrifuged at 4 °C and 1900 × g for 10 min. The supernatant was collected and then centrifuged at 12,000 × g at 4 °C for 20 min to remove cellular debris and impurities. Five hundred microlitres of serum from each group was diluted to 4 mL with prechilled DPBS buffer. The serum diluent for each tube was ultracentrifuged at 4 °C and 120,000 × g for 2 h, and the supernatant was discarded. Four millilitres of prechilled DPBS buffer was added to resuspend the precipitate. The suspensions were filtered through a 0.22 μm filter and ultracentrifuged at 4 °C and 120,000 × g for 70 min. The supernatant was discarded and then suspended in prechilled PBS. The protein content of serum exosomes in the mice with TAA determined using a BCA Protein Assay kit (Merck, USA).

### Characterization of exosomes

Twenty microlitres of exosome sample was adsorbed onto copper mesh. The morphology and size of the exosomes were observed with a HT-7700 transmission electron microscope (Hitachi, Japan), and the exosomes were subjected to nanoparticle tracking analysis (NTA) with a ZetaView S/N 17–310 system (Particle Metrix, Germany). Specific markers for exosomes, including Alix, GM130, CD9 and CD63 (Cell Signaling Technology, USA), were detected by Western blot analysis according to routine procedures.

### Sample preparation for LC‒MS/MS

For LC‒MS/MS analysis, exosomes were lysed with 8 mol/L urea (Sigma, USA), and proteins were extracted via centrifugation at 12,000 × g and 15 °C for 20 min. Protein concentrations in exosomes were quantified with a BCA protein assay kit. Small molecules in the protein samples were removed with 10 kD centrifugal filters (Sartorius Stedim Biotech GmbH, Germany), and then the protein samples were suspended in 100 μL of 100 mmol L^−1^ NH_4_HCO_3_ and 5 mmol/L dithiothreitol (DTT). After being incubated at 56 °C for 30 min, the samples were added to iodoacetamide (IAA) to achieve a final concentration of 20 mmol/L and shaken for 30 min in the dark. Protein was digested by adding trypsin (Promega, USA) to the filter (trypsin:protein ratio of 1:50) at 37 °C for 16 h, and enzyme-digested peptides were collected by centrifugation at 14,000 × g and 15 min for 25 °C. Finally, peptides were desalted with a C18 column (Agela Technologies, USA) and quantified according to the kit's instructions.

### LC‒MS/MS analyses

The exosomal peptides were analysed by using an EASY-nLC1200 connected to an Orbitrap fusion mass spectrometer (Thermo Scientific, Waltham, MA, USA) in data-dependent acquisition (DDA) mode. The exosomal peptides were subjected to gradient elution by using 5 ~ 100% acetonitrile (ACN) with 0.1% formic acid (FA) at 300 nL/min for 60 min. The elution process was as follows: 0 ~ 3 min (5% ACN), 5 ~ 13 min (5% ~ 10% ACN), 13 ~ 48 min (10% ~ 30% ACN), 48 ~ 55 min (30% ~ 100% ACN) and 55 ~ 60 min (100% CAN). The ion source voltage threshold was set to 2.1 kV for data-dependent acquisition (DDA). The DDA regimen included a first-order mass spectrometer survey scan at a resolution of 120,000 full width at half maximum (FWHM) (at 200 m/z) from 350 m/z to 1500 m/z with the automatic gain control (AGC) set to 2.0E5 (maximum injection time of 50 ms), followed by maximum-speed data acquisition at a resolution of 30,000 FWHM with the AGC set to 5.0E4.

### Protein identification

The original DDA data were searched against the UniProt database of *Mus musculus* (downloaded on September 22, 2022) using Proteome Discoverer v2.2.2.21. The product ion spectra and precursor were searched under initial mass tolerance values of 10 ppm and 0.02 Da, respectively. Trypsin digestion was selected, and up to two digestion sites were allowed to be missed. The fixed modification was set to aminomethylation of cysteine (57.02 Da), and the variable modification was set to oxidation of methionine (15.99 Da). The Q value used for protein identification in PD software was less than 0.01. The original DDA result file was imported into the MaxQuant software to generate a spectral library with a Q cut-off value of 0.99.

### Cluster analysis

*P* value < 0.05 and fold change (FC) > 1.5 were used as screening criteria for the selection of differentially expressed proteins (DEPs). A heatmap was constructed to visually display the expression trends of DEPs in different samples and then aggregate them into classes according to the existence of identical or similar expression trends to further compare the expression patterns of DEPs in serum exosomes between the sham group and the BAPN combined with Ang II-induced TAA model group.

### Bioinformatics analysis

Gene Ontology (GO) annotation analysis was employed to explore and analyse DEPs based on biological processes, molecular functions, and cellular components. Pathway analyses were performed to explore the pathways significantly enriched among DEPs according to KEGG (http://www.genome.jp/KEGG/).

## Statistical analysis

In this study, SPSS 23.0 software was used to perform statistical analyses for all data, and the values are expressed as the mean ± SD (mean ± SD). The data were tested for homogeneity of variance and then subjected to multiple comparisons between groups. Student’s t test was used to compare differences between two groups. Differences for which *p* was < 0.05 were considered statistically significant.

## Results

### The mouse model construction of TAA dependent on BAPN combined with Ang II

We constructed an animal model of TAA with BAPN plus Ang II treatment to explore the roles of serum exosomes in TAA. In this study, we found that the hair of the mice became dry and dull, and the activity level, diet, and drinking water consumed decreased in the model group. However, this phenomenon was not observed in the control and sham groups.

**Fig. 1:**
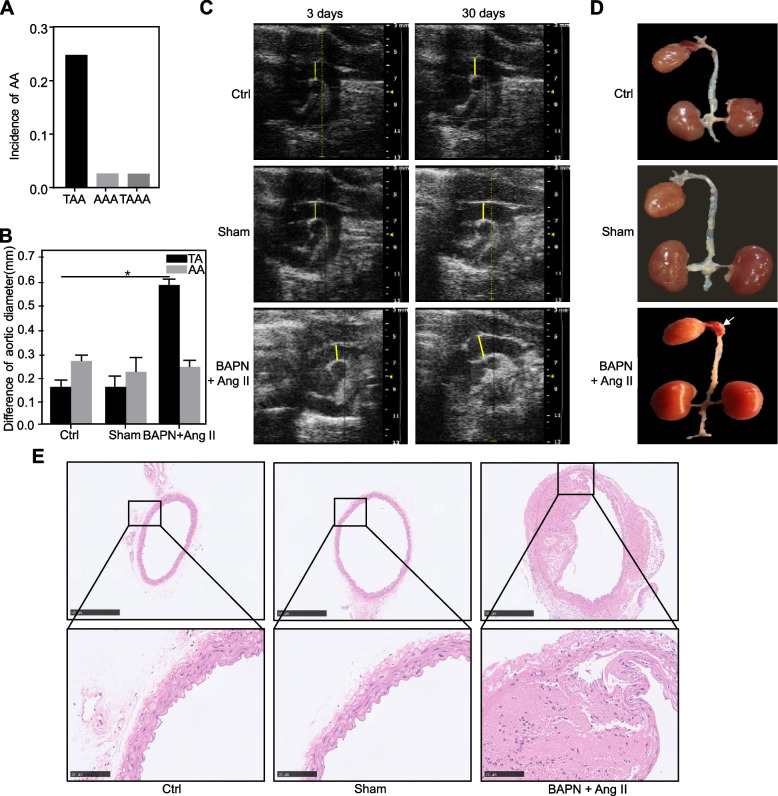
The construction of a TAA animal model in C57BL/6 mice. **A** The incidence of aortic aneurysm in the BAPN plus Ang II group. **B** The differences in aortic diameter among the three groups. The yellow lines indicate the measurement position. **C** The diameter of the thoracic aorta in the three groups was measured by echocardiography. **D** Anatomical images of the thoracic aorta of mice in the three groups; the white arrow indicates a TAA site. **E** HE staining of the thoracic aorta in the three groups of mice. *TAA*, Thoracic aortic aneurysm, *AAA* Abdominal aortic aneurysm, *TAAA* Thoracoabdominal aortic aneurysm

The results showed that administration of BAPN (1 g/d/kg) plus Ang II (1 μg/kg/min) induced TAA in C57BL/6 mice. After forty-eight hours of pumping Ang II, an echocardiogram showed that the inner diameter difference of the thoracic aorta in the model group was 1.5 times larger than that in the control group; this was defined as successful model construction. Approximately 25% of mice developed TAA, and the incidence of thoracoabdominal aortic aneurysm (TAAA) was 2.78% (Fig. 1A). In this group, 44.44% of the mice died of aortic dissection or rupture. However, no mice in the control group or sham group died. The aortic diameter was measured by echocardiography on the third day and the 30th day for the control group, sham operation group, and model group. There was no local tumour-like dilation of the thoracic aorta in the control group or sham group. The difference in the inner diameter of the thoracic aorta measured by echocardiography on the third day and the 30th day of control group and sham group mice was 0.156 ± 0.028 mm and 0.156 ± 0.043 mm, respectively. However, the inner diameter of the thoracic aorta of the model group mice was 0.569 ± 0.023 mm. The changes in the internal diameter of the abdominal aorta for the control group, sham group, and model group were 0.261 ± 0.024 mm, 0.217 ± 0.058 mm, and 0.237 ± 0.027 mm, respectively (Fig. 1B). The echocardiogram indicated that the thoracic aorta was globularly dilated in C57BL/6 mice in the BAPN combined with Ang II group (Fig. 1C).

We dissected the aortic vessels of mice using a stereomicroscope and found that the vessels of the ascending aorta and the aortic arch were obviously dilated and spherical. Intramural haematomas (IMHs) were observed in the lesions. However, the aortic wall was smooth without dilation in the control group and sham group (Fig. 1D). Haematoxylin and eosin staining showed that the vascular intima of the model group was damaged and discontinuous. In the control group and the sham group, the intima of the aortic wall was intact, and the vascular smooth muscle cells were arranged in a ring-like shape (Fig. 1E). Therefore, we generated a reliable and convenient TAA model in C57BL/6 mice to study the pathological process of TAA and explore therapeutic targets.

### Characteristics of serum exosomes from mice with TAA

**Fig. 2:**
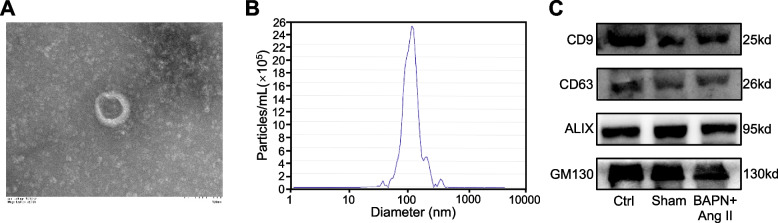
Characterization of exosomes. (**A**) TEM image of exosomes. (**B**) NTA of exosomes. (**C**) Western blot detection of CD63, CD9, GM130 and ALIX was performed after exosome protein extraction

To identify the characteristics of serum exosomes in mice with TAA induced by BAPN combined with Ang II, we isolated serum exosomes from C57BL/6 mice via ultracentrifugation. Transmission electron microscopy revealed similar morphological characteristics of exosomes, with round or elliptical vesicles and a double-layer membrane structure, among the control group, the sham group, and the model group (Fig. 2A). NTA of the isolated exosomes showed that the main peak of the vesicle size distribution was 122 ± 55.6 nm (Fig. 2B). Western blotting showed that the exosome markers CD63, CD9, GM130, and ALIX were all expressed (Fig. 2C). The above results indicated that exosomes were successfully isolated for further proteomic analysis.

### Mass spectrometry-based identification of proteins in serum exosomes from mice with TAA

**Fig. 3:**
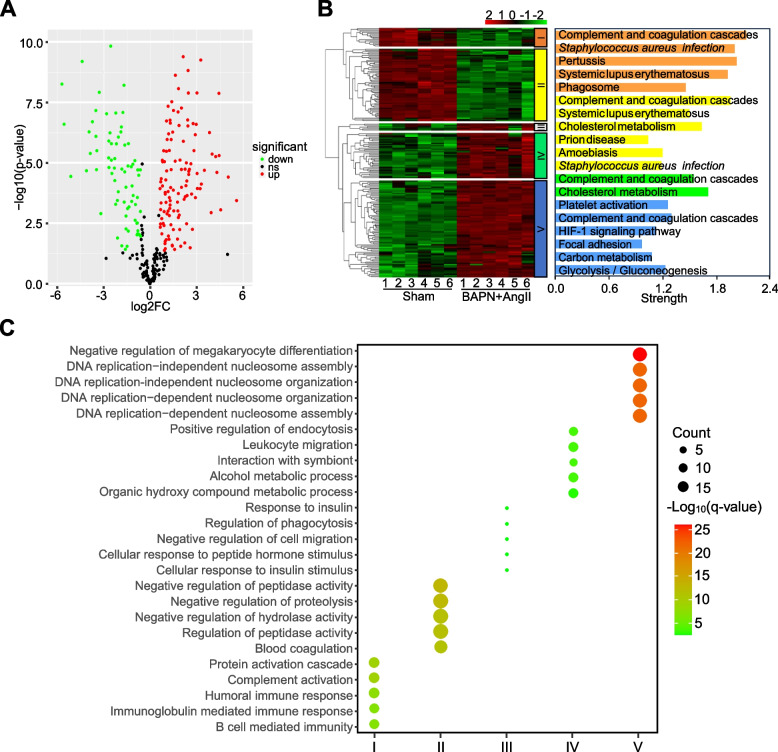
Comparison of DEPs in serum exosomes from mice with TAA induced by BAPN combined with Ang II. **A** Volcano plots were generated to show the DEPs in serum exosomes from mice with TAA induced by BAPN combined with Ang II. Red and green dots represent up- and downregulated proteins, respectively. **B** Heatmap analysis was performed to show the significant DEPs in mouse serum exosomes between the sham group and BAPN plus Ang II group. Red represents high expression, while green represents low expression. KEGG pathway analysis was performed to investigate the pathways significantly enriched in expressed proteins. **C** Functional classification of the dysregulated proteins was performed by GO enrichment analysis

A mass spectrometry-based proteomics study was used to characterize the protein constituents of exosomes during TAA development induced by BAPN plus Ang II. A total of 399 proteins were identified by proteome analysis of serum exosomes from mice in the sham group and the model group. To further illustrate the differences in protein expression, we generated volcano plots for the sham and model groups by considering proteins with *p* < *0.05* and a fold change (FC) greater than 1.5 to be significantly dysregulated (up- or downregulated) compared to the sham group. Compared with that in the sham group, the expression of 196 proteins significantly differed in serum exosomes of mice with TAA. There were 122 significantly upregulated DEPs and 74 significantly downregulated DEPs (Fig. 3A). Clustering analysis revealed the relationships among the proteins in the sham group and the model group. In this study, we found that in the serum exosomes of mice in the sham group and the model group, there were five representative patterns of differential protein expression (Fig. 3B). GO enrichment analysis was carried out to identify exosome proteins based on biological process and molecular function. In cluster I, we found that the proteins were mainly involved in biological processes such as the *protein activation cascade*, *complement activation*, *humoral immune response*, and *immunoglobulin-mediated immune response*. The biological process GO terms *negative regulation of peptidase activity*, *negative regulation of proteolysis*, and *blood coagulation* were mainly enriched in cluster II. Cluster III proteins were involved in biological processes such as *cellular response to insulin stimulus* and *negative regulation of cell migration*. In cluster IV, the most markedly enriched GO terms for biological process were *leukocyte migration* and *positive regulation of endocytosis*. *DNA replication* and *negative regulation of megakaryocyte differentiation* was the most significantly enriched GO term for the biological process in the cluster V proteins (Fig. 3C). Therefore, exosomal proteins are likely to be involved in the pathophysiological process associated with aortic aneurysm.

### Functional analysis of upregulated DEPs in TAA

**Fig. 4:**
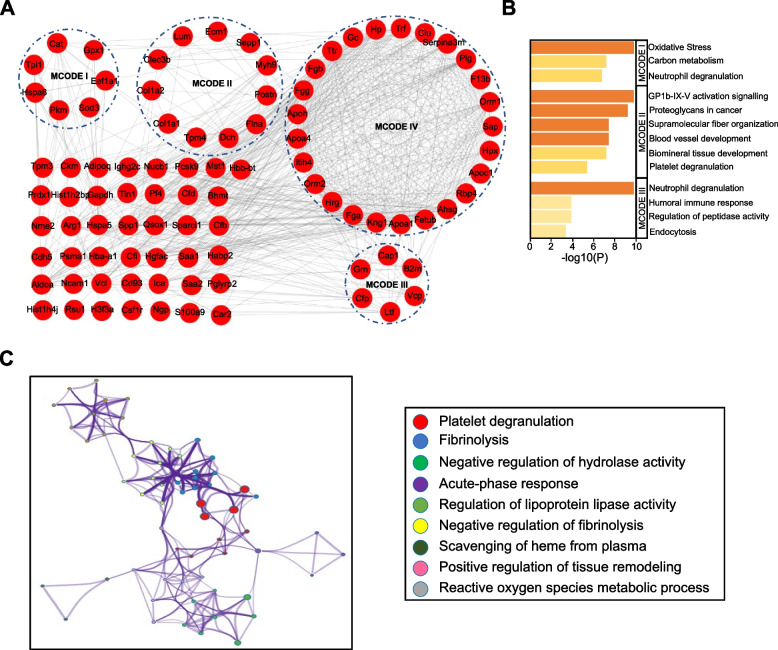
Protein network analysis of upregulated DEPs in mouse serum exosomes between the sham group and BAPN plus Ang II group. (**A**) STRING analysis revealed the protein–protein interaction (PPI) network of the 122 upregulated proteins. (**B**), (**C**) Functional analysis based on Metascape was performed to investigate significantly enriched terms by the upregulated proteins in the MCODE I-III (**B**) and MCODE IV (**C**), respectively

GO enrichment analysis was employed to analyse differentially upregulated proteins in the serum exosomes of mice with TAA induced by BAPN plus Ang II. The results indicated that the molecular functions of the upregulated proteins were mainly enriched in *signaling receptor binding*, *molecular function regulation*, and *antioxidant activity*. These proteins were closely related to the *stress response*, *protein metabolism process regulation*, *inflammatory response*, and *regulation of the immune system process*. After performing reactome pathway analysis of serum exosome proteins of mice with TAA, we observed that *platelet activation*, *signaling and aggregation* signaling pathways, *plasma lipoprotein assembly*, *remodeling and clearance* signaling pathways, and *smooth muscle contraction* signaling pathways were distinctly enriched. The KEGG pathway enrichment analysis indicated that the *complement and coagulation cascades* were significantly enriched in the upregulated proteins (Table 1). We constructed a protein‒protein interaction (PPI) network for 122 significantly upregulated proteins in serum exosomes from TAA model mice in comparison with the sham group and screened the core cluster MCODE I-IV in the PPI network based on the Cytoscape app with the MCODE plugin (Fig. 4A). By performing functional analysis of the core clusters, we found that multiple process terms were related to the development of TAA (Fig. 4B, C). In these interaction networks, the upregulated proteins of the MCODE I complex were involved in *oxidative stress* (Cat, Gpx1, Sod3) (Fig. 4B). The upregulated proteins of the MCODE II complex participated in *blood vessel development* (Ecm1, Myh9, Postn, Flna, Tpm4) and *supramolecular fibre organization* (Col1a1, Col1a2, Dcn, Clec3b) (Fig. 4B). Our analysis showed that four proteins were involved in the *humoral immune response* (B2m, Cfp) and *regulation of peptidase activity* (Vcp, Grn) (Fig. 4B). Most proteins were involved in *fibrinolysis* (Trf, Plg, Clu, F13b, Sap, Fga, Fgb, Fgg) and *acute-phase response* (Hp, Hpx, Rbp4, Orm1, Orm2, Itih4, Ttr, Gc) and were highly connected within the MCODE IV complex (Fig. 4C). This indicates that TAA formation is regulated by multiple biological processes associated with upregulated exosomal proteins.

**Table 1:** Functional enrichment analysis of up-regulated proteins

Enrichment Analysis	Annotation	Protein Species	FDR Values
BP	Response to stress	52	1.17E-17
BP	Regulation of immune system process	27	2.47E-10
BP	Regulation of protein metabolic process	36	1.33E-08
BP	Regulation of signal	35	2.44E-07
BP	transduction	13	3.76E-06
MF	Inflammatory response	27	3.72E-08
MF	Signaling receptor binding	27	6.32E-08
MF	molecular function regulator	14	9.7E-08
MF	Enzyme inhibitor activity	7	3.22E-06
MF	Enzyme regulator activity	17	9.27E-06
KEGG	Complement and coagulation cascades	10	1.71E-09
KEGG	HIF-1 signaling pathway	4	0.0127
KEGG	Thyroid hormone synthesis	3	0.0338
KEGG	ECM-receptor interaction	3	0.0376
KEGG	Vitamin digestion and absorption	2	0.0376
Reactome	Platelet activation, signaling and aggregation	25	1.69E-24
Reactome	Plasma lipoprotein assembly, remodeling, and clearance	4	0.0018
Reactome	Smooth muscle contraction	3	0.0018
Reactome	Platelet adhesion to exposed collagen	2	0.0065
Reactome	Crosslinking of collagen fibrils	2	0.0153

Note: Biological Process (BP); Molecular Function (MF)

### Functional analysis of downregulated DEPs in TAA

**Fig. 5:**
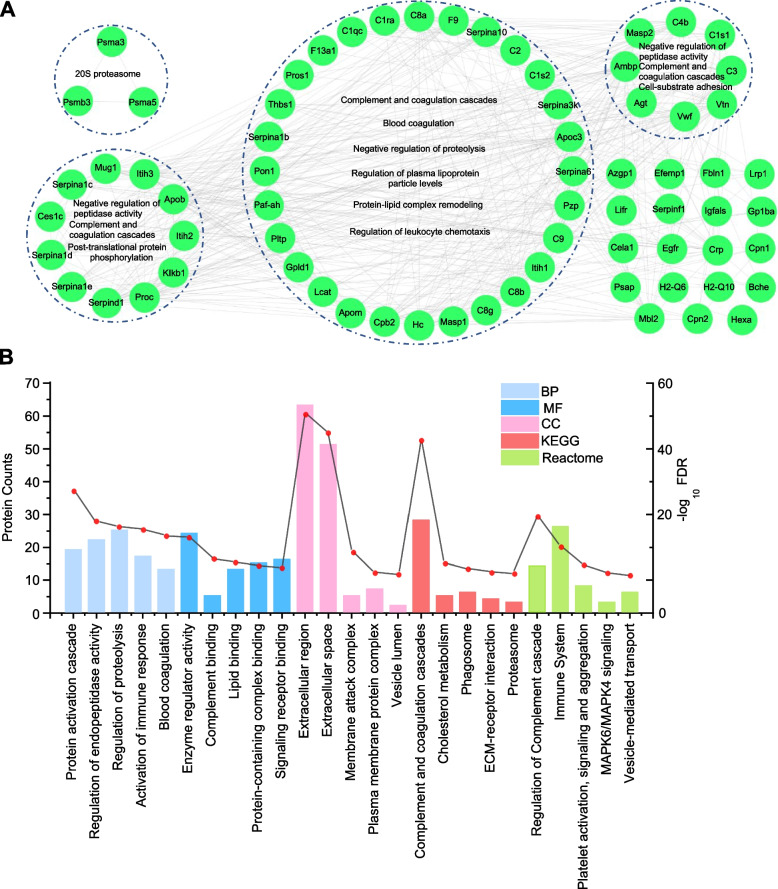
Bioinformatics analysis of downregulated DEPs. **A** STRING analysis revealed the protein–protein interaction (PPI) network of the 74 downregulated proteins. **B** GO enrichment, KEGG pathway and Reactome pathway analyses were used to elucidate the potential biological functions of all downregulated proteins in serum exosomes of mice with TAA

In this study, we constructed a PPI network for 74 serum exosome proteins that were significantly downregulated compared with the sham group and screened core cluster MCODE I-IV by bioinformatics analysis. At the same time, we performed functional analysis of these four core clusters based on Metascape (http://metascape.org/gp/index.html#/main/step1). The analysis demonstrated that the significantly downregulated proteins in MCODE I-IV were involved in multiple biological processes that were related to the TAA process, such as *complement and coagulation cascades* (C8a, C2, C1s2, C9, C8b, C8g, C1qc), *blood coagulation* (F9, Serpina10, Serpina6, Pzp, Masp1, Hc, Cpb2, Pltp, Pla2g7, Serpina1b, Thbs1, Pros1, F13a1) and *regulation of plasma lipoprotein particle levels* (Apob, Apoc3, Apom) (Fig. 5A). Functional and pathway enrichment analyses were implemented to assess downregulated DEPs in serum exosomes from mice with TAA (Fig. 5B). Enrichment analysis indicated that the molecular function of the downregulated DEPs was related to *enzyme regulator activity* and *protein-containing complex binding activity* and was significantly involved in biological processes, such as *regulation of endopeptidase activity*, *regulation of proteolysis* and *blood coagulation*. Reactome pathway analysis demonstrated that these DEPs were mainly enriched in the *immune system*, *regulation of complement cascade*, *platelet activation*, *signaling and aggregation*, *MAPK6/MAPK4 signaling*, and *vesicle-mediated transport*. KEGG pathway analysis was also performed to investigate the pathways significantly enriched for these downregulated proteins. We found that the most enriched KEGG pathways were mainly the *complement and coagulation cascades* (Fig. 5B). Interestingly, downregulated exosomal proteins in multiple MCODE complexes were involved in the biological process *complement and coagulation cascade*, which suggests that the complement and coagulation cascade may play an important role in the development of aortic aneurysm.

## Discussion

Exosomes play an important role in cell-to-cell communication. Exosomes have been a hot topic in biological research in the past few decades. However, there have been few studies on exosomes in the context of TAA. Tong et al. demonstrated that exosomes derived from fibroblasts transfer angiotensin-converting enzymes to vascular smooth muscle cells, thereby increasing Ang II levels and activating angiotensin II type 1 receptors in vascular smooth muscle cells to promote cell migration [13]. Exosomes also play an important role in the formation of aneurysms. Previous studies demonstrated that the exosomes secreted by aortic fibroblasts cause short-term loss of miR-133a when the stress of the thoracic aortic vessel wall increases, which leads to changes in the phenotype of fibroblasts and further leads to vascular remodeling during the development of thoracic aneurysm [14, 15]. These data indicate that exosomes might play an important role in TAA, which provides a new perspective for further studies on the pathogenesis of TAA.

In the past few decades, technological advances in the field of proteomics have facilitated the identification of secreted proteins associated with various disease types, especially cancer [16]. Liquid chromatography-tandem mass spectrometry (LC‒MS/MS)-based proteomic analysis has facilitated the study of proteins secreted by multiple types of cells, which may be an effective strategy for identifying candidate diagnostic biomarkers and therapeutic targets [17]. In our study, we performed proteomics analysis of serum exosome proteins from mice with TAA induced by BAPN combined with Ang II to reveal the mechanisms of the disease. This is the first time proteome profiling has been utilized to investigate the changes in serum exosomal protein expression in mice with TAA induced by BAPN plus Ang II.

In the present study, we generated a volcano plot to analyse and visualize serum exosome protein expression profile changes between mice with TAA induced by BAPN combined with Ang II and mice in the sham group (Fig. 3A). Our current proteome analyses of the serum exosomes of mice with TAA induced by BAPN combined with Ang II resulted in the identification of 305 DEPs compared with the sham group. We used fold change (FC) > 1.5 and *P* < 0.05 as the screening criteria and identified a total of 196 proteins that were significantly up- or downregulated. Interestingly, only 37 DEPs have been reported in the context of aortic aneurysm. Most of them have been reported for abdominal aortic aneurysms, highlighting the knowledge gap concerning exosomes in TAA.

Multiple signalling pathways regulate the development of TAAs, and changes in exosomal proteins also control the development of TAAs. In our experiments, some proteins, such as Fibrinogen alpha (Fga), Fibrinogen beta (Fgb), Fibrinogen gamma (Fgg), Orosomucoid 1 (Orm1), Orosomucoid 2 (Orm2), Haptoglobin (Hp), and serum amyloid p-component (Sap), were significantly upregulated in serum exosomes from mice with TAA induced by BAPN combined with Ang II. Most of these proteins have been found to be involved in fibrinolysis and the acute-phase response. Furthermore, an in-depth analysis of these DEPs might be helpful for obtaining a more comprehensive understanding of the mechanisms of TAA. For example, we found 566-fold higher expression of Hp in exosomes derived from the serum of mice with TAA induced by BAPN combined with Ang II compared with those derived from mice in the sham group. Previous studies identified Hp as an acute-phase response glycoprotein whose expression is increased in serum exosomes in various diseases, such as inflammation and osteoarthritis (OA) [18, 19]. Hp is expressed in many cell types and tissues, such as macrophages, pulmonary epithelial cells, fibroblasts, and vascular smooth muscle cells [19, 20]. A previous study has also shown that haptoglobin is a chemotactic protein that promotes vascular smooth muscle cell (VSMC) migration and induces endothelial tube formation in the context of coronary collateralization [21]. Ruzevick et al. demonstrated that the number of macrophages in the aneurysm wall of transgenic Hp mice increased significantly, which suggest that Hp accelerate aneurysm formation by promoting inflammation. Moreover, the researchers indicated that transgenic Hp2-2 may be a strong independent predictor of rapid aneurysm growth [22].

Sap, which is also known as the serum amyloid p component, is a member of the pentraxin family that interacts with pathogens and cell debris, activating macrophages and neutrophils to clear them, and it also blocks colocalization in patients with atherosclerotic plaques [23]. We observed 4.4-fold upregulation of Sap in serum exosomes of TAA in the current study. Zheng et al*.* established an atherosclerosis model induced by a high-fat diet in Apo E^−/−^ and Sap^−/−^ mice and found that the formation of atherosclerosis was significantly inhibited in Sap^−/−^ mice. Further study have demonstrated that vascular endothelial cells induce chronic inflammation and secrete cytokines and cell debris during the development of atherosclerosis [24]. Increased Sap sensitivity promotes the polarization of macrophages to the M1 phenotype. M1-type macrophages turn into foam cells via phagocytosis of cytokines and cell debris and then accumulate between the vascular intima and media to accelerate the development of atherosclerosis. Previous study have shown that apolipoprotein C1 (ApoC1) overexpression in ApoE^−/−^ mice can exacerbate atherosclerosis by promoting severe hypertriglyceridemia, increasing very low density lipoprotein (VLDL) synthesis and reducing lipid residue clearance by lipoprotein lipase, which may be related to the pathogenesis of AAA. Immunofluorescence also confirmed the overexpression of ApoC1 in the mural thrombus in the aorta of AAA mice, suggesting that the intramural thrombus may be an important source of ApoC1 [25]. Previous study demonstrated that in atherosclerotic ApoE^−/−^ mice, ApoC1 was involved in the pathogenesis of AAA by promoting macrophage polarization towards the M1 phenotype, increasing cholesterol outflow, and promoting foam cell formation [26]. In addition, Poulsen et al*.* identified the apolipoprotein family as Sap ligands by affinity pull-down assays and coimmunoprecipitation [27]. Bus et al. discovered that the number of M1 macrophages in the glomerular arteries of apolipoprotein C1 (ApoC1) transgenic mice increased, which indicated that ApoC1 expression induce glomerulosclerosis by increasing the cytokine response of macrophages [28]. Therefore, during the development of atherosclerosis, ApoC1 and Sap in exosomes may synergistically promote the polarization of macrophages to the M1 phenotype. M1 macrophages accelerate foam cell formation via binding of phagocytic inflammatory cells to endothelial cell factors, and cell debris accelerates both foam cell formation and accumulation between the vascular intima and media, leading to the development of atherosclerosis, which further leads to the formation of TAA.

To understand the proteome profile of serum exosomes, GO enrichment and KEGG pathway analysis were used to analyse significant DEPs to understand the molecular mechanisms involved in the progression of TAA. Our study showed that response to stress, regulation of immune system process, and regulation of protein metabolic process were highly enriched, indicating that these biological processes may play a key role in the development of TAA. Previous study have shown that plasma-derived exosomes from peripheral arterial disease patients induce VSMC migration via immune regulation [29]. Notably, KEGG pathway analysis revealed that complement and coagulation cascade pathways were both enriched in significantly up- and downregulated proteins. Related research demonstrated that Ficolin-3 in microvesicles isolated from activated platelets from AAA patients could be involved in complement-coagulation crosstalk, and high Ficolin-3 levels were positively correlated with aortic diameter [30]. Based on the results of the PPI analysis, proteins with strong interactions with multiple downregulated proteins are mainly involved in the complement and coagulation cascades, which further indicates that complement-coagulation processes play an important role in TAA. Interestingly, we found that von Willebrand factor (vWF) was 20.9-fold downregulated in serum exosomes of mice with TAA compared with sham mice, which is inconsistent with the results of previous studies [31]. Thus, further studies with more experiments and larger clinical sample sizes are warranted.

There are several limitations of this study. First, we used animal models to study the pathogenesis of TAA, which may not accurately represent the pathophysiology of human TAA. Second, our conclusions are based solely on the results of bioinformatics analysis. Therefore, clinical sample and further experiments are required to verify those conclusions in future research.

## Conclusions

In summary, the current study is the first to reveal the protein profile of serum exosomes in mice with TAA induced by BAPN combined with Ang II. Our data identified several key candidate proteins in serum exosomes, such as Hp and Sap, which may be potential biomarkers for TAA development.

### Supplementary Information

**Additional file 1. **

## Data Availability

The datasets used and analyzed during the current study are available from the corresponding authors on reasonable request.
