# Supplementary material for: Label-free quantitative proteomic analysis of serum exosomes in mice with thoracic aortic aneurysm
Source: Proteome Sci. 2023 Oct 24;21:19. doi: 10.1186/s12953-023-00220-x (PMC10594717; doi:10.1186/s12953-023-00220-x)
Supplement: Supplementary file 1 — Additional file 1. [file 12953_2023_220_MOESM1_ESM.docx]

**Original western blot for three repeats**

Repeat1

Repeat2

Repeat3

ALIX(95kd)


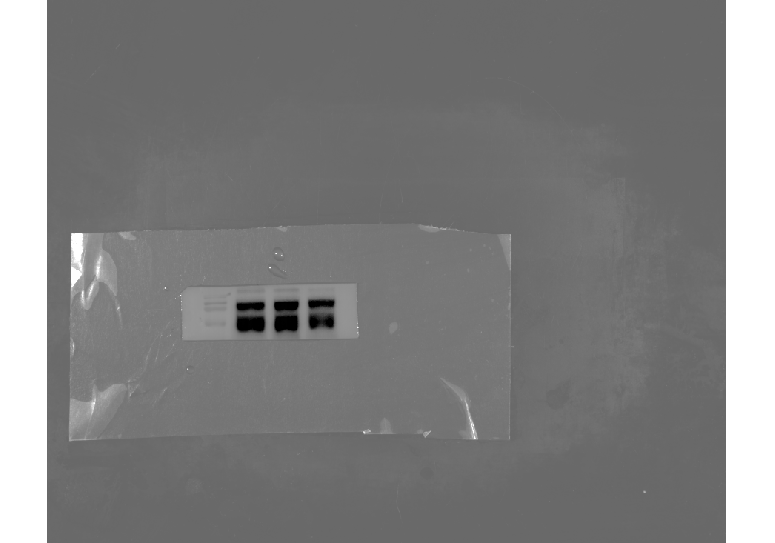


130

70

100

170


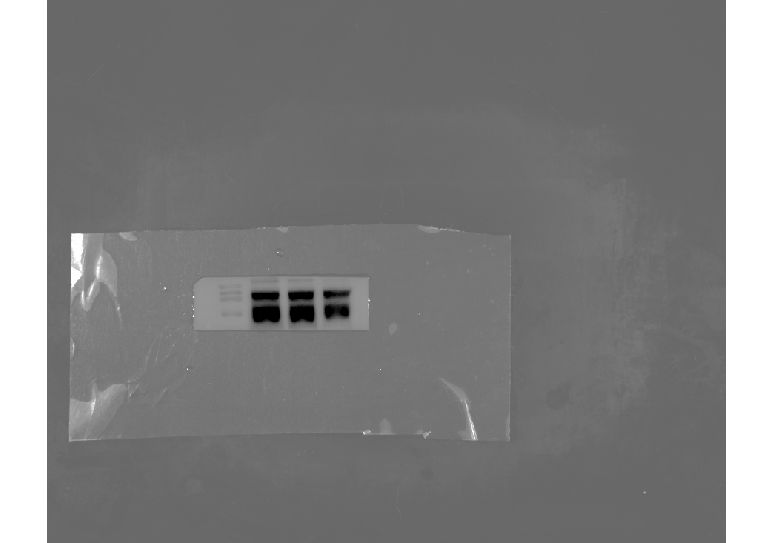


130

70

100

170


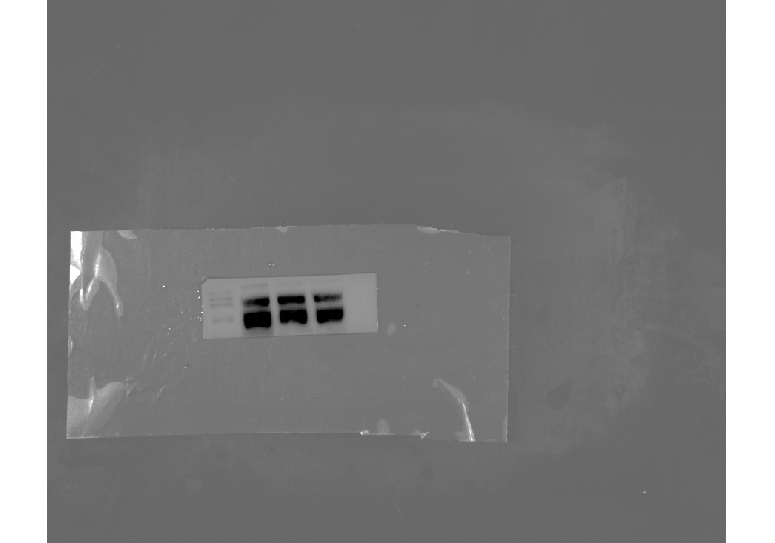


130

70

100

170

CD63(26kd)


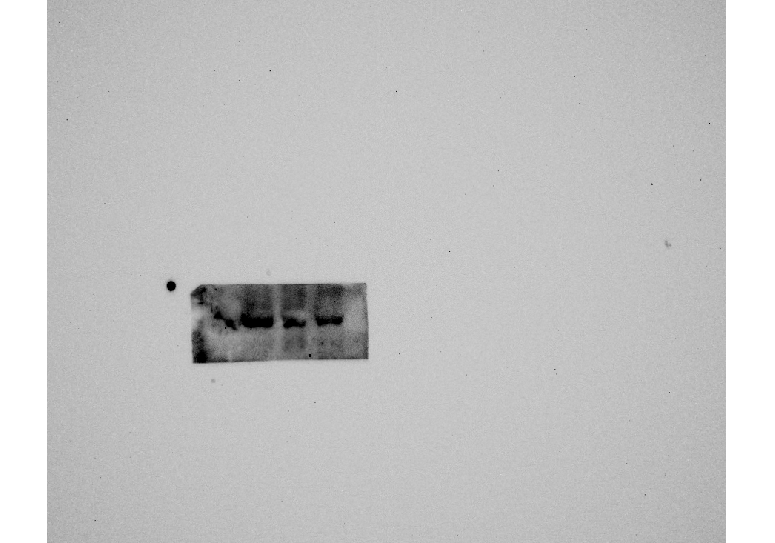


CD9(25kd)


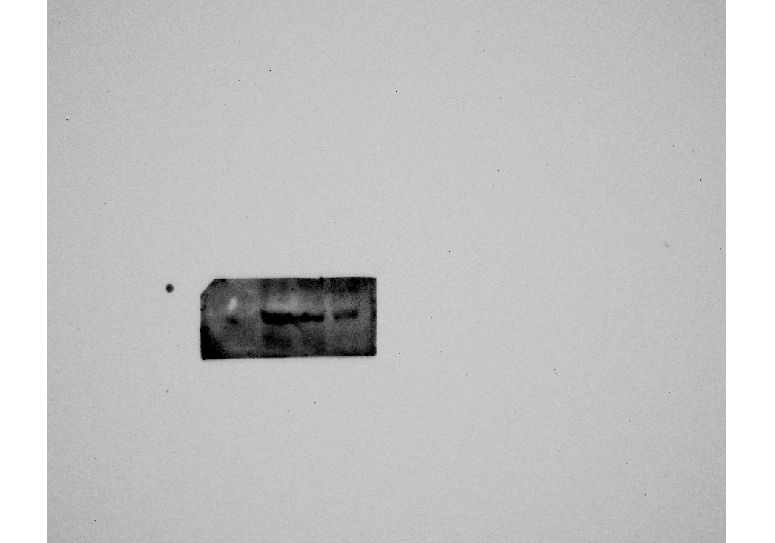

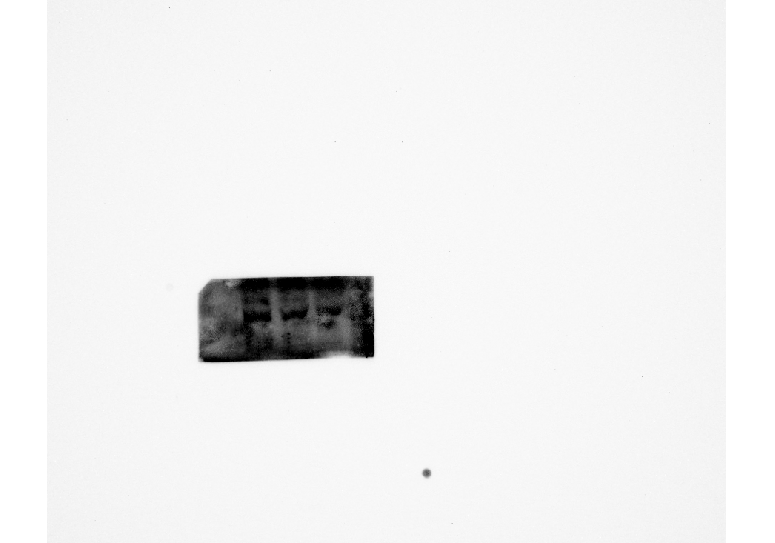

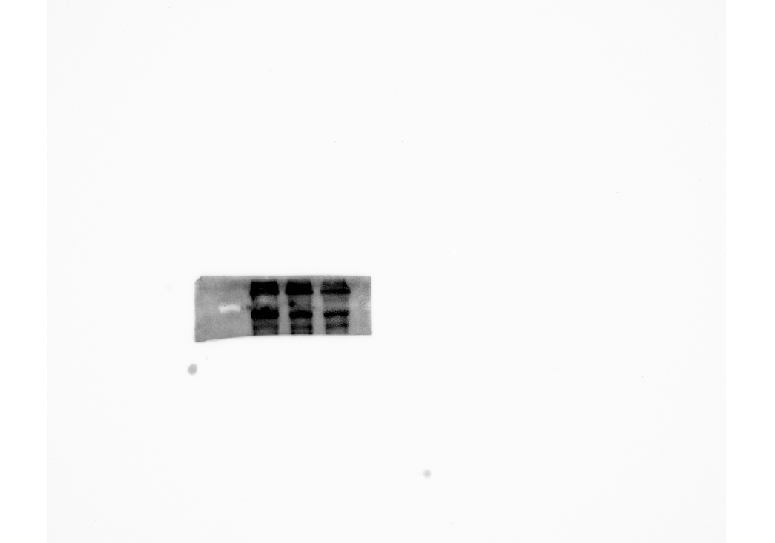


70


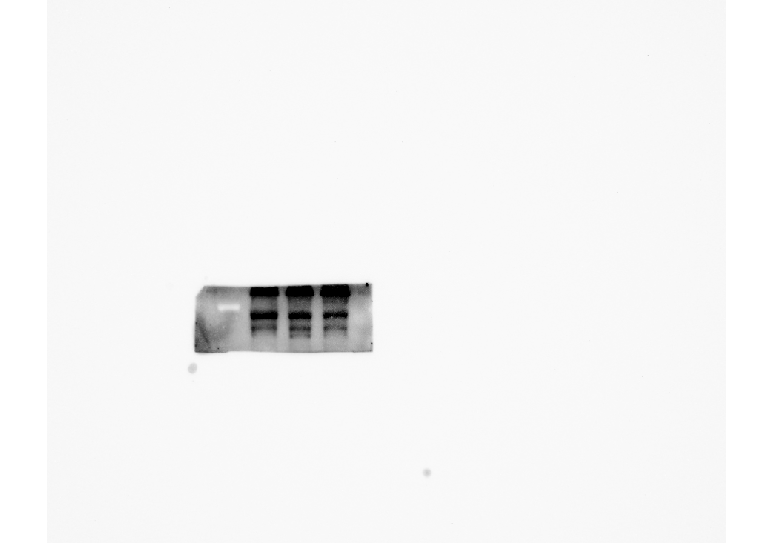


70


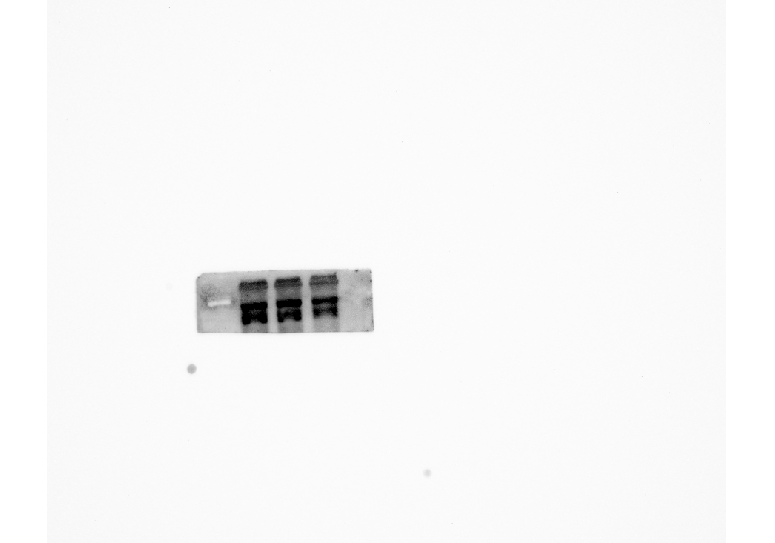


70

GM130(130kd)


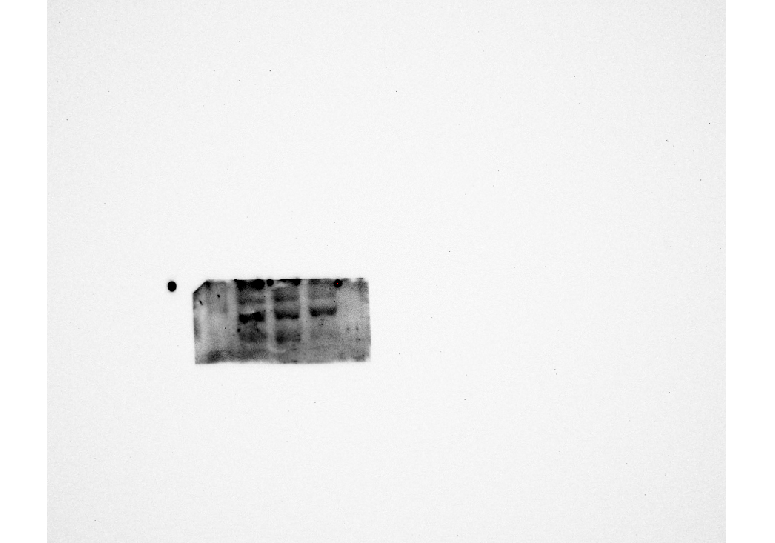


Ctrl

Sham

BAPN+AngII


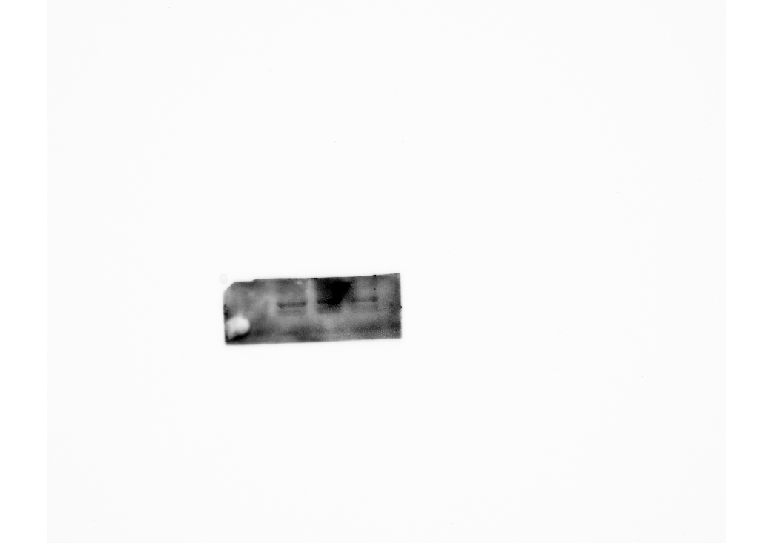


Ctrl

Sham

BAPN+AngII


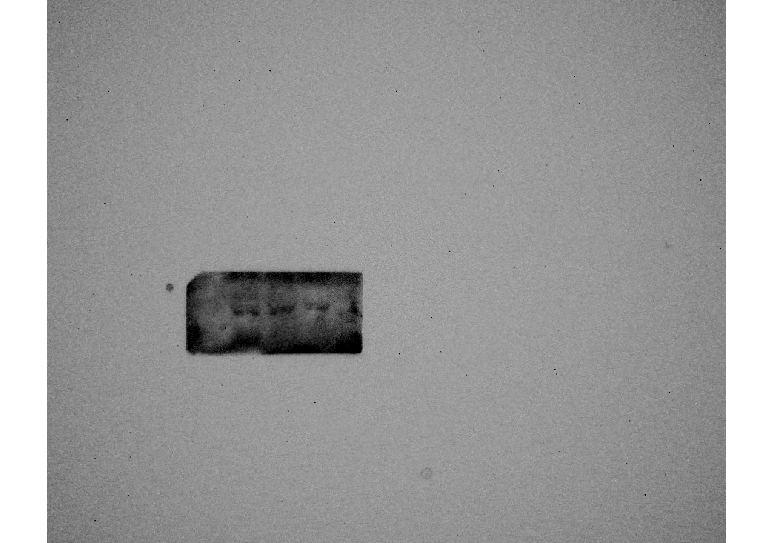


Ctrl

Sham

BAPN+AngII
